# An unusual case of gardening ocular injury during Covid-19 lockdown

**DOI:** 10.1016/j.ijscr.2021.02.005

**Published:** 2021-02-05

**Authors:** Riccardo Nocini, Giorgio Giampaoli, Dario Bertossi

**Affiliations:** a G.B. Rossi University Hospital AOUI Borgo Trento, Department of Surgery, Dentistry, Paediatrics and Gynaecology, University of Verona, Verona, Italy; b G.B. Rossi Hospital and Medical University of Verona, Department of Surgery, Dentistry, Paediatrics and Gynaecology, University of Verona, Piazzale L.A. Scuro 10, 37134, Verona, Italy

**Keywords:** Eye, Domestic accident, Gardening, Ocular

## Abstract

**Introduction:**

Facial trauma are an important cause of serious ocular morbidity. In particular domestic trauma are a small part of total.

COVID-19 pandemic has been influencing our life in a way never seen before, people need to remain at home due to lockdown restrictions. In this scenario we are seeing an increase in the percentage of domestic facial trauma. In other hand pandemic has influenced the possibility of hospitalization, so daily based procedures increased their importance in global treatment planning.

**Case presentation:**

A 58 yo man presented to our ward with a foreign body in left eyebrow. Trauma happened during gardening.

**Clinical discussion:**

The importance of imaging to perform the right procedure has become more important during pandemic to reduce time of hospitalization.

**Conclusion:**

CT scan and ophtalmology consult have been the guideline to avoid a more invasive treatment which was performed in an outpatient regimen with local anesthesia.

## 1 Introduction

Facial trauma is an important cause of serious ocular morbidity. Each year, there are 55 million eye injuries globally that restrict patient activities for more than 1 day whereas 19 million have at least unilateral permanent vision loss and 1.6 million people get one eye blinded by their injury [1] 9,5% of these facial injuries are due to gardening activities [2].

At the current time most of the world countries are in lockdown due to Corona virus pandemic and very few activities are allowed. Among these, gardening in private garden is still allowed.

## 2 Case presentation

This is a case report of a 58-year-old man. He had no history of past diseases, he referred to take any therapy, he denied allergies to medicine, inhalants and food.

He presented to our department with a metallic nail embedded in his left upper eyelid while gardening after 1 month lockdown. He was cutting grass when a nail, accidentally found in the floor, penetrated his left eyebrow.

**Fig. 1:**
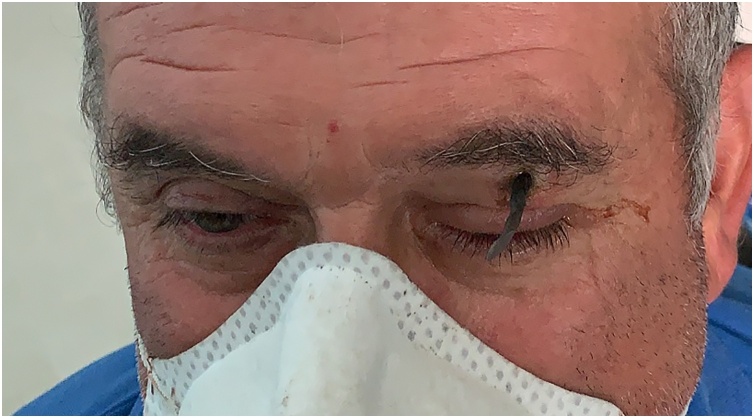
Frontal vision of patient.

**Fig. 2:**
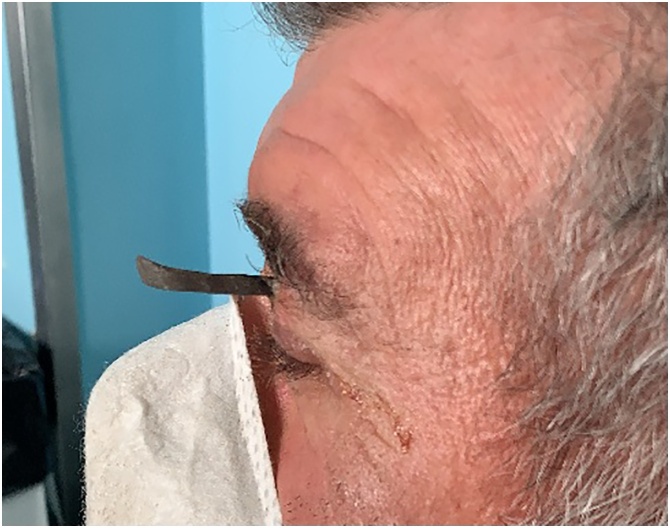
Lateral vision of patient.

On initial examination, his left eyelid was locked by the foreign body and opened with difficulty therefore limited evaluation could be performed (Figs. 1, 2 ).

**Fig. 3:**
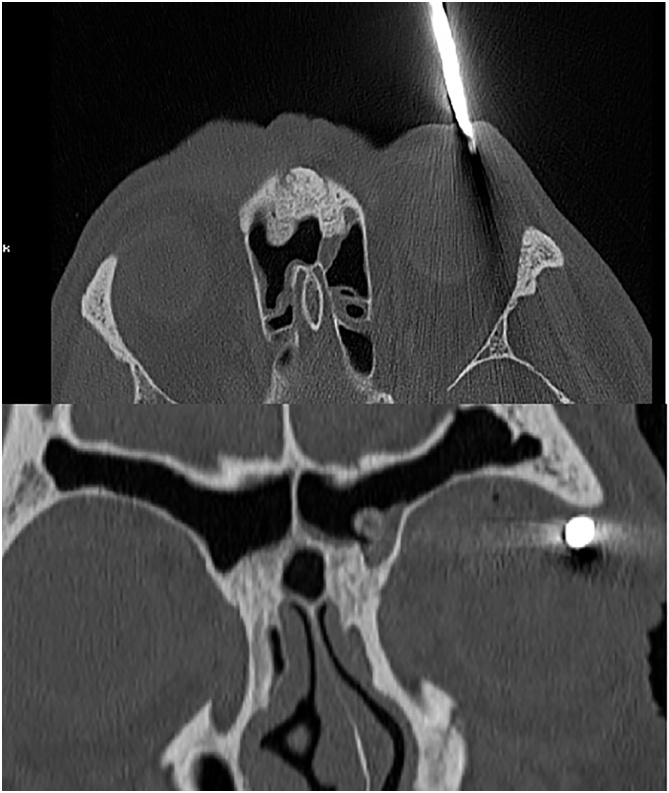
Orbit CT-Scan.

**Fig. 4:**
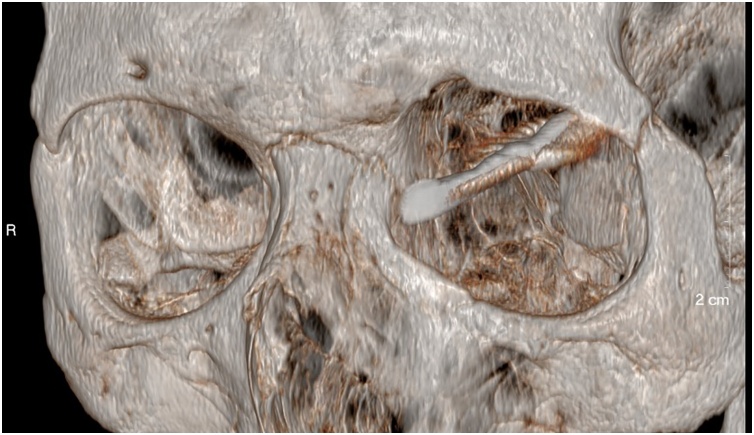
3D reconstruction of orbit CT-Scan.

An orbital CT scan was performed, and it showed the nail luckily didn’t touch the eye globe nevertheless it had penetrated the superior rectus muscle and the lateral rectus muscle. There was no involvement of the bony orbit (Figs. 3, 4 ).

An ophthalmologic evaluation was requested to check visual acuity and to assess a regular extrinsic eye movement. They found limitation of the ocular motility with restriction in looking upward, morover a limitation in the eyelid movement was founded.

We therefore planned the surgical treatment. After meticulous cleaning followed by disinfection of the upper lid and of the surrounding area with Iodine solution, we injected subdermally and 0,5 mm deep into the eye cavity a local anesthesia (0.5cc of carbocaine with adrenaline 1:100.000) and the foreign body was extracted with caution making a rotation of 35 degrees in clockwise and anticlockwise rotation but maintaining in an orthogonal position in order to not injury the eyeball. After a deep cleansing with saline solution, medication with iodine gauze was applied. Oral azytromicine 500 mg a day for 6 days was prescribed and local skin tobramicine ointment was applied.

**Fig. 5:**
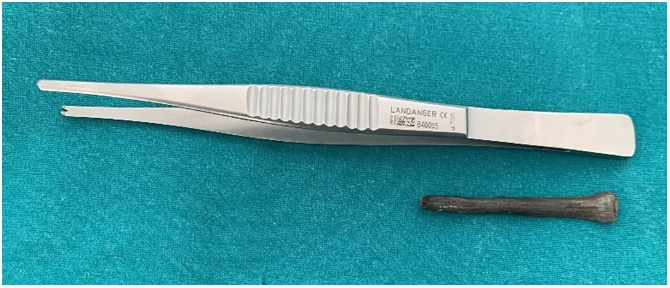
The nail after extraction.

**Fig. 6:**
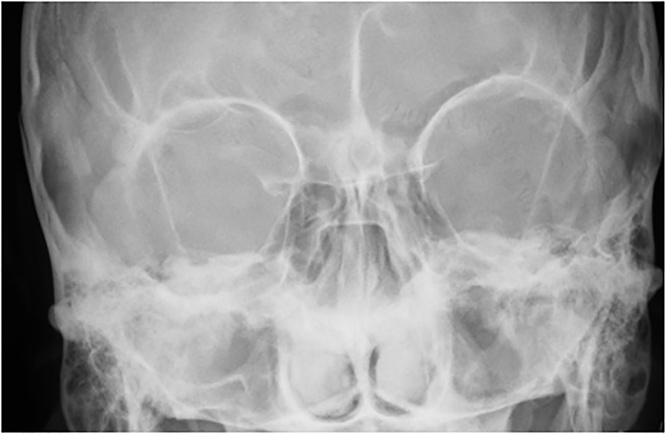
Orbit X-Ray after extraction.

An X-Ray check was performed to assess any residual other accidental foreign body to confirm the accuracy of extraction (Figs. 5, 6 ).

Patient was checked for follow-up at 3−7-15 days. At 30 days ophtalomologic assessment confirmed 100 % visual acuity and muscular normal function.

All the procedures were made in consensus with SCARE guidelines [3]

## 3 Discussion

Covid-19 had an important influence in CMF activities, in particular a diminishing in global activities was seen [4,5].

According to literature, in our experience in Verona Hospital, we saw a general diminishing in facial trauma due to aggression, car and job accident. In 2019 from march to may we admitted and operated 41patients for facial trauma, in the same period in 2020 during lockdown we admitted and operated only 6 patients for the same reasons. Despite of this, we have been seeing an increase in trauma due to domestic activities or accident for example gardening, domestic falling, recreational activities. (2019 8patients 19,5% of total VS 2020 4patients 66 % of total).

Covid-19 imposed to us a prioritization in hospitalization and, where possible, a different managing in procedure (ex local anesthesia procedure) [6].

In particular case of CMF trauma, examination (physical and radiological) and ophtalmology consult are the essential base to perform the correct diagnosis to plan the right treatment.

In case of pandemic, that phase acquire a more important role.

In standard condition (no pandemic), that case need hospitalization with an observation period of 24/48 h, but fortunately with the accuracy of inspection and diagnostic it could be performed in local anesthesia without post-op observation.

Procedures that involving orbit need particular attention [7,8] and surely and individual planning.

## 4 Conclusion

In time of Coronavirus there is an increase of domestic facial trauma. This case report show how we manage cases of foreign bodies after gardening without hospitalization.

CT scan and ophtalmology consult have been the guideline to avoid a more invasive treatment which was performed in an outpatient regimen with local anesthesia.

## Declaration of Competing Interest

The authors report no declarations of interest.

## Funding

The authors whose names are listed above certify that they have no affiliations with, or involvement in, any organization or entity with any financial interest (such as honoraria; educational grants; participation in speakers’ bureaus; membership, employment, consultancies, stock ownership, or other equity interest; and expert testimony or patent-licensing arrangements) or nonfinancial interest (such as personal or professional relationships, affiliations, knowledge, or beliefs) in the subject matter or materials discussed in this manuscript.

## Ethical approval

All procedures performed in studies involving human participants were in accordance with the ethical standards of the institutional and/or national research committee and with the 1964 Helsinki Declaration and its later amendments or comparable ethical standards.

## Consent

Written informed consent was obtained from the patient for publication of this case report and accompanying images. A copy of the written consent is available for review by the Editor-in-Chief of this journal on request.

## Author contribution

Riccardo Nocini: Writing paper, data collection and analysis.

Giorgio Giampaoli: Writing paper, data collection and analysis, study concept.

Dario Bertossi: Writing paper, data collection and analysis, study concept.

## Registration of research studies

Not applicable.

## Guarantor

Dr. Giorgio Giampaoli.

## Provenance and peer review

Not commissioned, externally peer-reviewed.
